# SSD-YOLO: a lightweight network for rice leaf disease detection

**DOI:** 10.3389/fpls.2025.1643096

**Published:** 2025-08-18

**Authors:** Canlin Pan, Shen Wang, Yahui Wang, Chaoyang Liu

**Affiliations:** ^1^ School of Information Engineering, Henan Institute of Science and Technology, Xinxiang, China; ^2^ School of computer and information engineering, Xinxiang University, Xinxiang, China; ^3^ School of Mechanical and Electrical Engineering, Henan Institute of Science and Technology, Xinxiang, China

**Keywords:** disease identification, object detection, deep learning, YOLOv8, attention mechanism

## Abstract

Rice leaf diseases significantly impact yield and quality. Traditional diagnostic methods rely on manual inspection and empirical knowledge, making them subjective and prone to errors. This study proposes an improved YOLOv8-based rice disease detection method (SSD-YOLO) to enhance diagnostic accuracy and efficiency. We introduce the Squeeze-and-Excitation Network (SENet) attention mechanism to optimize the Bottleneck structure of YOLOv8, improving feature extraction capabilities. Additionally, we employ a Dynamic Sample (DySample) lightweight dynamic upsampling module to address high similarity between rice diseases and backgrounds, enhancing sampling accuracy. Furthermore, Shape-aware Intersection over Union (ShapeIoU) Loss replaces the traditional Complete Intersection over Union (CIoU) loss function, boosting model performance in complex environments. We constructed a dataset of 3000 rice leaf disease images for experimental validation of the SSD-YOLO model. Results indicate that SSD-YOLO achieves average detection accuracies of 87.52%, 99.48%, and 98.99% for rice brown spot, rice blast, and bacterial blight respectively—improving upon original YOLOv8 by 11.11%, 1.73%, and 3.81%. The model remains compact at only 6MB while showing significant enhancements in both detection accuracy and speed, providing robust support for timely identification of rice diseases.

## 1 Introduction

Rice, as one of the world’s staple food crops, faces significant threats from various diseases, resulting in substantial economic losses. Rice diseases predominantly manifest on leaves, and their diagnosis currently relies heavily on expert experience and visual assessment. This approach is not only inefficient but also prone to misjudgment, thereby impacting the effectiveness and accuracy of disease management. Given the numerous challenges associated with identifying rice leaf diseases, there is an urgent need to develop more scientific and objective diagnostic methods to enhance both the precision and efficiency of identification.

The rapid advancement of information technology has facilitated closer integration between agriculture and IT. Leveraging innovative tools such as computer vision, machine learning, and deep learning algorithms, agricultural informatization is advancing towards greater intelligence, providing robust support for agricultural production. In the realm of crop disease identification, drones equipped with image acquisition devices enable more efficient collection of disease images. Identification methods have evolved from rule-based machine learning algorithms to data-driven deep learning algorithms, shifting the reliance on expert experience for feature extraction to automated processing, which significantly improves the efficiency and accuracy of identification.

In recent years, deep learning models have demonstrated outstanding performance in numerous tasks, particularly convolutional neural networks (CNNs) which have achieved breakthroughs in computer vision. CNN-based models offer end-to-end processing capabilities, automatically learning and extracting low-level features, enabling non-experts to perform crop disease diagnosis using computer vision (Liu et al., 2018; Sun et al., 2021). However, CNN models typically have a large number of parameters and high computational costs. Most CNN models used for diagnosing rice leaf diseases require high-performance GPU cards for acceleration, limiting their practicality in field applications (Agarwal et al., 2020). Therefore, optimizing these models’ computational complexity and parameter count is essential to enhance their usability and deployment convenience in real-world environments.

In the field of crop disease and pest identification, while CNNs have been widely applied, single-image classification methods alone cannot meet practical application requirements. Beyond identifying disease types, it is crucial to obtain detailed information such as the number of infected leaves and their distribution areas. To address this, object detection technology has emerged, achieving precise localization of disease regions alongside classification, thus providing more comprehensive diagnostic results.

Deep learning-based object detection methods are primarily categorized into single-stage and two-stage approaches. Single-stage algorithms like SSD (Liu et al., 2016) and the YOLO series (Bochkovskiy et al., 2020; Jocher et al., 2021; Li et al., 2022) are better suited for real-time applications due to their speed, whereas two-stage algorithms like R-CNN (Girshick et al., 2014) and Fast R-CNN (Girshick, 2015) are more complex. Among these, the YOLO series has achieved remarkable success. To meet the specific needs of rice disease detection, this study proposes an improved model based on YOLOv8 called SSD-YOLO. This model incorporates the SENet attention mechanism to optimize the Bottleneck structure, enhancing feature extraction capabilities. Additionally, it introduces a DySample lightweight dynamic upsampling module to focus sampling points on target areas while ignoring background elements, addressing the issue of high similarity between diseases and the background. Furthermore, ShapeIoU Loss replaces the original CIoU loss function, improving the model’s detection performance in complex environments.

The remainder of this paper is organized as follows: Section 2 reviews related works on traditional machine learning methods, lightweight CNNs, and attention mechanisms in crop disease detection. Section 3 details the proposed SSD-YOLO methodology, including improvements to YOLOv8’s backbone network (SENetV2), DySample upsampling, and ShapeIoU loss function. Section 4 presents experimental results, covering dataset construction, evaluation metrics, comparative analyses with other models, and ablation studies. Finally, Section 5 concludes the study and discusses future research directions.

## 2 Related works

### 2.1 Traditional machine learning methods

Traditional approaches to identifying plant diseases and pests predominantly utilize image feature extraction techniques. Specifically, methods such as Histogram of Oriented Gradients (HOG) (Dalal and Triggs, 2005), Scale-Invariant Feature Transform (SIFT) (Lowe, 2004), and Speeded Up Robust Features (SURF). (Bay et al., 2006) are employed to extract salient features from images of affected plants. These extracted features subsequently serve as inputs for training classifiers, including Support Vector Machines (SVM) (Cortes and Vapnik, 1995) and k-Nearest Neighbor (k-NN) (Wang and Hodges, 2005), thereby enabling accurate classification of various types of diseases and pests. (Chaudhary and Kumar, 2024) propose an advanced rice disease detection method combining Gray-level Co-occurrence Matrix (GLCM) and Intensity-Level Based Multi-Fractal Dimension (ILMFD) for feature extraction, demonstrating 96.7% accuracy for brown spot detection using SVM classifier, outperforming ANN and Neuro-GA approaches in identifying major rice diseases including leaf blast and bacterial blight. (Jamjoom et al., 2023) developed an SVM-based image processing system for plant disease detection, utilizing GLCM and LBP features to identify four diseases (Phytophthora infestans, Fusarium graminearum, Puccinia graminis, and tomato yellow leaf curl) with 97.2% accuracy. The proposed method systematically processes images through acquisition, pre-processing, segmentation, feature extraction, and classification stages, demonstrating superior performance over manual detection approaches. (Sahu and Minz, 2023) proposed an advanced plant disease detection system combining AFKMRG segmentation with Enhanced LSTM classification, optimized through FSJ-FOA, achieving 98.35% accuracy and 98.40% precision in multi-disease identification. The method significantly improved upon traditional techniques by adaptively fusing region-growing segmentation with evolutionary algorithm-enhanced feature extraction and classification.

However, traditional machine learning methods face several limitations, including the necessity for manual feature extraction and selection, which is both time-consuming and requires specialized knowledge. Additionally, these methods lack robust tuning mechanisms and suffer from lower computational efficiency. Consequently, in certain applications, these drawbacks have led to the gradual adoption of deep learning techniques as a more effective alternative.

### 2.2 Lightweight convolutional neural network

Deep learning is a subfield of machine learning, with convolutional neural networks (CNNs) being one of its prominent models. CNNs are primarily employed for image processing and computational vision tasks such as image classification and object detection. Common object detection models include YOLO (Redmon et al., 2016) and Faster R-CNN (Ren et al., 2017). (Abulizi et al., 2025) propose DM-YOLO, an improved YOLOv9-based method for tomato leaf disease detection, which integrates DySample for small lesion feature extraction and MPDIoU for overlapping lesion localization, achieving higher precision (92.5%) and mAP (86.4%) compared to baseline models. (Li et al., 2025) propose YOLO v5s-ours, an enhanced potato defect detection model integrating Coordinate Attention (CA), Adaptive Spatial Feature Fusion (ASFF), and Atrous Spatial Pyramid Pooling (ASPP), which achieves 85.1% mAP (a 13.7% improvement over baseline) while maintaining real-time performance (30.7 fps), enabling practical automated sorting of defects like greening, rot, and mechanical damage. (Huang et al., 2025) propose GDS-YOLO, an enhanced YOLOv8n-based rice disease detection model incorporating GsConv for efficiency, Dysample for feature preservation, SCAM for background suppression, and WIoU v3 for precise localization, achieving 4.1% higher mAP50 with 23% fewer parameters compared to baseline, demonstrating effective feature extraction for complex rice disease identification. (Feng et al., 2025) develop LCDDN-YOLO, an efficient cotton pest detector combining DSConv and BiFPN with CBAM attention, achieving 6.5% higher mAP@50 than YOLOv8 at reduced computational costs (12.9% fewer parameters), enabling real-time disease monitoring in resource-constrained field environments.

These studies collectively demonstrate that lightweight CNN models have exhibited superior performance in rice disease identification and object detection tasks, significantly enhancing detection accuracy and processing efficiency.

### 2.3 Attention mechanism

(Zhu et al., 2025) propose an improved YOLOv8-based model for grape leaf disease detection, incorporating Spatial Pyramid Dilated Convolution (SPD-Conv) and an Efficient Multi-Scale Attention (EMA) Module, which achieves 96.17% AP (a 1.13% improvement over YOLOv8) while maintaining a compact model size (7.1 MB), significantly enhancing small-target recognition accuracy for diseases like black rot. (Wang and Liu, 2025) propose TomatoGuard-YOLO, an advanced lightweight framework based on YOLOv10, which integrates Multi-Path Inverted Residual Units (MPIRU) for enhanced multi-scale feature extraction and a Dynamic Focusing Attention Framework (DFAF) for adaptive region prioritization, achieving state-of-the-art performance (94.23% mAP50 at 129.64 FPS) with an ultra-compact model size (2.65 MB), offering a transformative solution for intelligent tomato disease detection systems. (Wang et al., 2025) propose RGC-YOLO, an efficient multi-scale rice pest detection model based on YOLOv8n, which integrates RepGhost for feature reuse, GhostConv for computational efficiency, and CBAM for enhanced feature extraction, achieving 93.2% mAP50 with 33.2% fewer parameters and 21.3% faster inference on embedded devices compared to baseline, enabling real-time monitoring in field conditions. (Kumar et al., 2023) propose a Multi-scale YOLOv5 network with DenseNet-201 backbone and Bidirectional Feature Attention Pyramid Network (Bi-FAPN) for early-stage rice disease detection, achieving 94.87% accuracy and 0.71 IoU while reducing computational costs through principled pruning, demonstrating superior performance on the RLD dataset compared to existing methods. (Li et al., 2024) propose GhostNet_Triplet_YOLOv8s, an enhanced YOLOv8s model incorporating GhostNet for lightweight backbone architecture and Triplet Attention for precise disease localization, achieving 91.40% mAP@0.5 with 50.2% model size reduction and 43.1% lower FLOPs compared to baseline, successfully deployed on a WeChat mini-program for real-time maize disease detection.

In summary, the addition of attention mechanisms can significantly improve model performance, enabling it to focus more effectively on important features and enhancing the model’s interpretability.

### 2.4 Comparison of methods and existing problems

The existing rice disease detection methods, as shown in **Table 1**, can be classified into three technical routes: (1) Feature engineering methods based on traditional machine learning (such as SVM+GLCM) can achieve an accuracy of 96.7%, but rely on manual feature design; (2) Lightweight CNN models (such as DM-YOLO and GDS-YOLO) increase mAP to 92.5% through structural optimization (DySample+MPDIoU), but have insufficient sensitivity for small object detection; (3) Attention mechanism enhanced models (such as EMA-YOLO, RGC-YOLO) combined with CBAM/EMA modules can achieve mAP50 up to 96.17%, but model complexity and lesion background similarity (>0.7) remain the main bottlenecks. The current method achieves a good balance between parameter size (2.65MB~7.1MB) and inference speed (30~129 FPS), but still needs to break through the dynamic feature decoupling of edge blur (IoU loss>15%) and small lesions (<5px) unique to rice leaf lesions. These bottleneck problems provide clear directions for model optimization.

**Table 1:** Comparison of existing disease detection methods.

Method type	Representative models	Key improvements	Feature
Traditional ML	SVM + GLCM/ILMFD	Combines GLCM and multi-fractal dimension features	Manual feature engineering, poor generalization
	AFKMRG + Enhanced LSTM (Sahu and Minz, 2023)	Adaptive region-growing segmentation with FSJ-FOA optimization	High computational complexity
Lightweight CNN	DM-YOLO	DySample + MPDIoU	Low sensitivity to small lesions
	YOLO v5s-ours	CA attention + ASFF + ASPP	High real-time demand (30.7 fps)
	GDS-YOLO	GsConv + DySample + SCAM	23% fewer parameters
Attention Mechanisms	EMA-YOLOv8	SPD-Conv + EMA	Model size: 7.1MB
	TomatoGuard-YOLO	MPIRU + DFAF	Ultra-lightweight (2.65MB)
	RGC-YOLO	RepGhost + CBAM	Optimized for embedded devices
Multi-Scale Fusion	Bi-FAPN-YOLOv5	DenseNet-201 + Bidirectional Feature Attention Pyramid	Requires model pruning
	GhostNet_Triplet_YOLOv8	GhostNet + Triplet Attention	50.2% smaller model size

## 3 Method

### 3.1 YOLOv8

YOLOv8 is the next major update version of YOLOv5, which was open-sourced by Ultralytics on January 10, 2023. YOLOv8 inherits the achievements of the previous YOLO series and has been widely applied in multiple fields such as image classification, object detection, and instance segmentation. When YOLOv8 was released, five different-sized network models were introduced, marked as n (nano), s (small), m (medium), l (large), and x (extra-large). The number of parameters and computational cost of these models increase successively, and their accuracy also improves accordingly.

**Figure 1:**
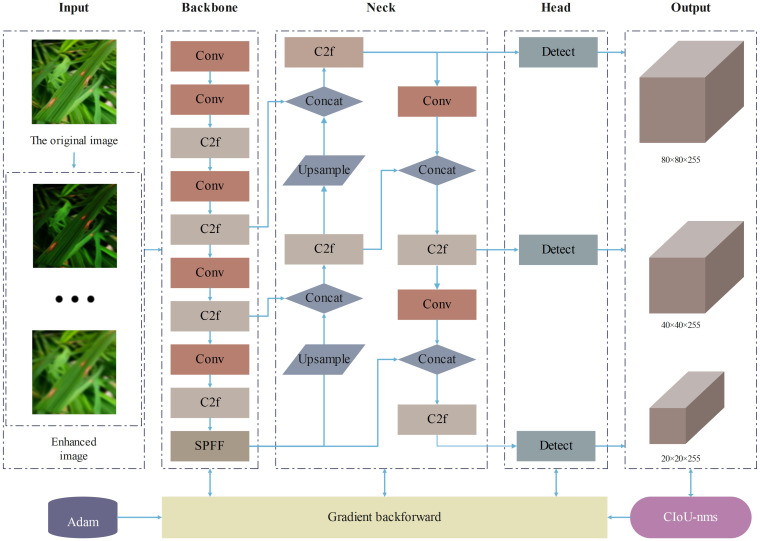
YOLOv8 architecture diagram.

Considering the resource constraints of mobile and embedded devices and the demand for lightweight models, this study selected the YOLOv8n model with the smallest number of parameters and computational cost. As shown in **Figure 1**, the main structure of YOLOv8 consists of three parts: the backbone network, the neck network, and the head network. In the backbone network part, YOLOv8 adopts the C2f structure with richer gradient flow, which is mainly responsible for feature fusion. It can effectively fuse feature maps of different levels, enabling the model to obtain more abundant information, reduce information loss, and significantly enhance the model’s ability to recognize image content. The design of the C2f module enables YOLOv8 to more effectively capture and utilize various details in the image, thereby improving the accuracy of object detection. In the neck network part, YOLOv8 adopts the C2f module and Path Aggregation Network (PANet) structure for feature aggregation, layer by layer aggregating information from shallow to deep, further enhancing the feature expression ability. In the head network part, YOLOv8 adopts the mainstream decoupled head structure, separating the classification and detection heads, and introduces the Anchor-Free strategy. Through these improvement strategies, YOLOv8 has made significant progress in loss calculation and network structure, enhancing the overall performance of the model.

### 3.2 Improve the backbone network

Squeeze-and-Excitation Network(SENet) (Hu et al., 2020) is a channel-wise attention mechanism that enhances feature representation by recalibrating channel-wise feature responses. In 2023, Mahendran introduced Squeeze-and-Excitation Network Version 2 (SENetV2), which integrates the squeeze-and-excitation operations with the multi-branch architecture of ResNeXt, thereby improving the granularity of feature expression and the integration of global information. The C2f module in YOLOv8’s neck network employs two convolutional layers, a Split operation, concatenation, and Bottleneck blocks to facilitate feature extraction and fusion, thereby enhancing the model’s feature representation capabilities. To further exploit the potential of feature expression, we incorporate SENetV2’s multi-branch structure and squeeze-and-excitation mechanism into the feature fusion process.

**Figure 2:**
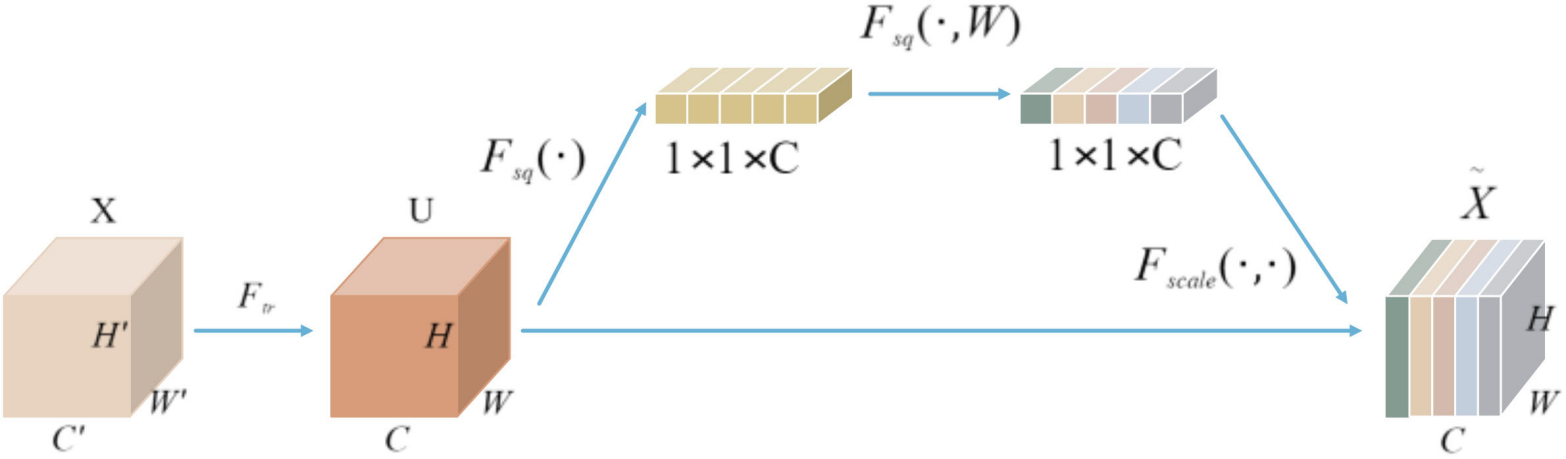
SENet module.

As illustrated in **Figure 2**, the SENet module applies global average pooling to compress the spatial dimensions of feature map U from H×W×C to a 1×1×C channel descriptor. This descriptor then undergoes two fully connected layers for nonlinear transformation, resulting in a set of channel-wise weights. These weights are subsequently multiplied element-wise with the original feature map U, producing a recalibrated feature map X. By increasing the sensitivity of each channel, SENet effectively emphasizes important features while suppressing less relevant ones.

**Figure 3:**
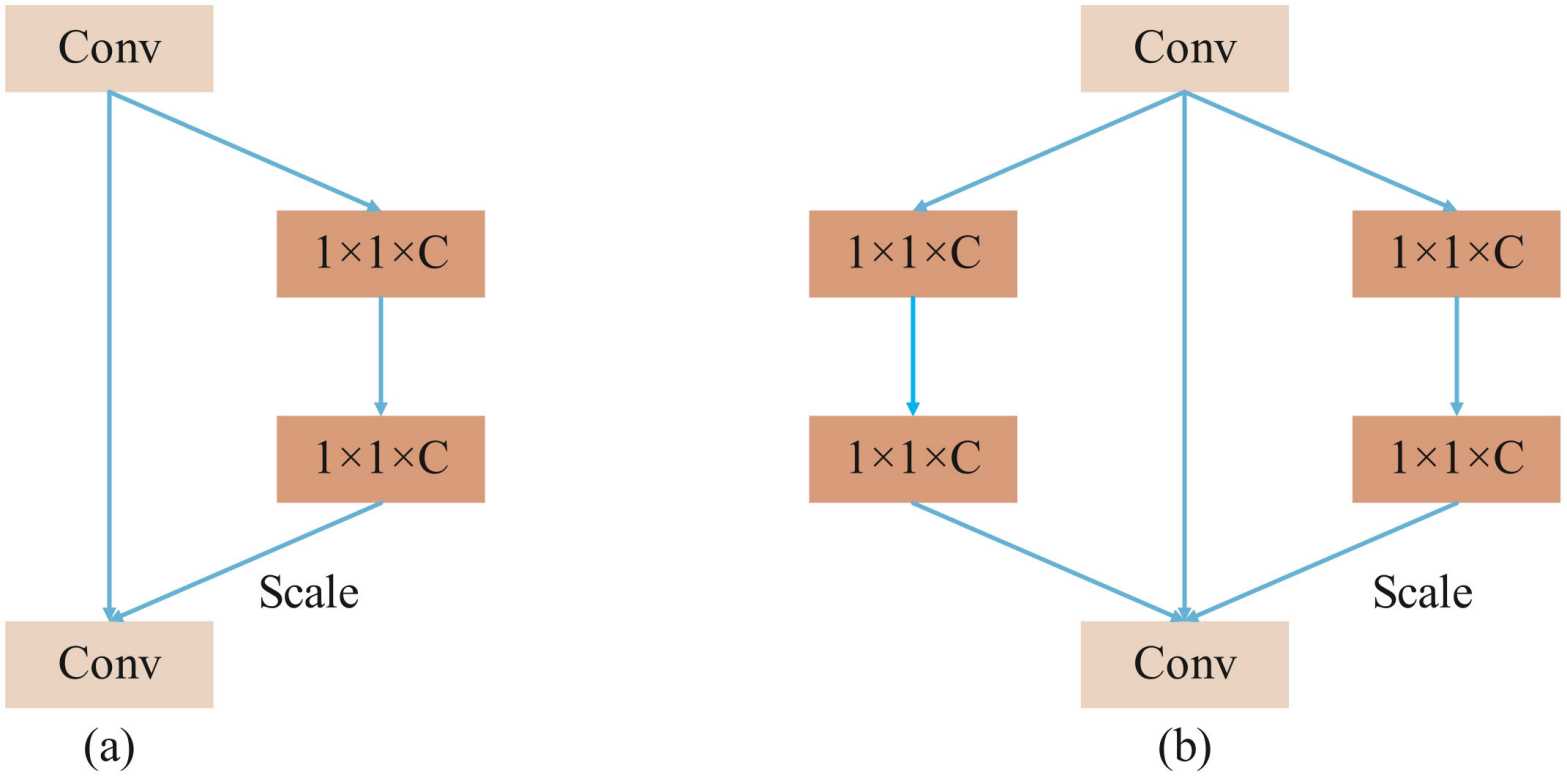
Comparison diagram of SENet and SENetV2 **(a)** SENet; **(b)** SENetV2.

SENetV2 integrates the squeeze-and-excitation operations from SENet with the multi-branch architecture of ResNeXt. It employs multi-branch fully connected layers for the compression and excitation steps, followed by feature scaling. This design slightly increases the model’s parameter count but significantly enhances its performance. **Figure 3** illustrates a comparison between the structures of SENetV2 and SENet. Both architectures can selectively transmit key features, but SENetV2 introduces increased cardinality between layers, aggregating feature information from multiple branches. Consequently, SENetV2 achieves richer and more diverse feature learning, making it better suited for representing complex features.

This study integrates the SENetV2 attention mechanism into the neck network of YOLOv8, which enables the network to capture diverse features from input data. This integration refines feature representation, enhances the model’s capability to extract disease characteristics in complex backgrounds, and consequently improves detection accuracy.

### 3.3 Introduce DySample

In the upsampling layer of YOLOv8, the commonly used method is nearest neighbor interpolation, which achieves upsampling by replicating the values of the nearest neighbor pixels. However, this method ignores the smooth transition characteristics between pixels, relies only on a few surrounding pixels for prediction, and overly focuses on spatial information while failing to fully consider the semantic information of the feature map. Additionally, the traditional upsampling process based on convolution kernels is usually accompanied by high computational complexity and parameter overhead, which contradicts the design goal of lightweight network architectures. Therefore, the DySample upsampling module (Liu et al., 2023) is introduced in the feature fusion step to replace the original upsampling method.

**Figure 4:**
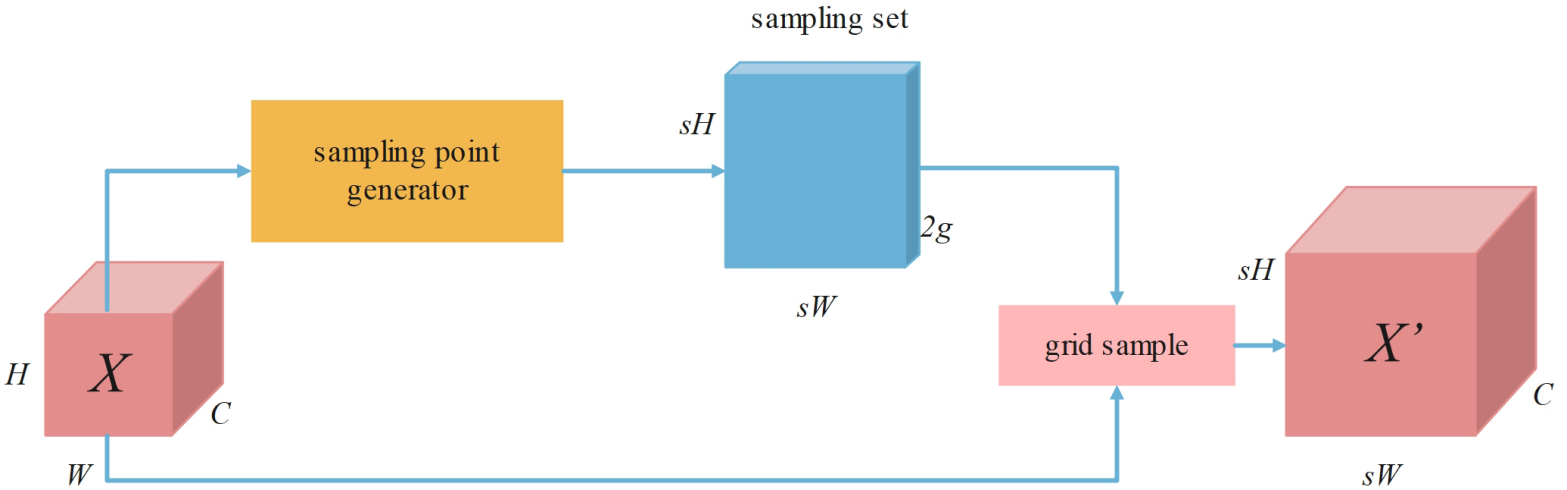
Upsampling process of Dysample.

DySample adopts a point sampling strategy, which can generate content-aware upsampling results in a simple and efficient way without the need for additional high-resolution feature maps as input. This approach not only helps maintain the model’s high performance but also significantly reduces model complexity and computational costs. The upsampling process of DySample is shown in **Figure 4**.

Given a feature map X of size C×H×W and a point sampling set S of 2g×sH×sW, where 2g represents the x and y coordinates, the grid_sample function re-samples X using the positions in the point sampling set S, generating a feature map X’ of size C×sH×sW, as shown in Equation 1:


(1) X'=Gridsample(X,S)

**Figure 5:**
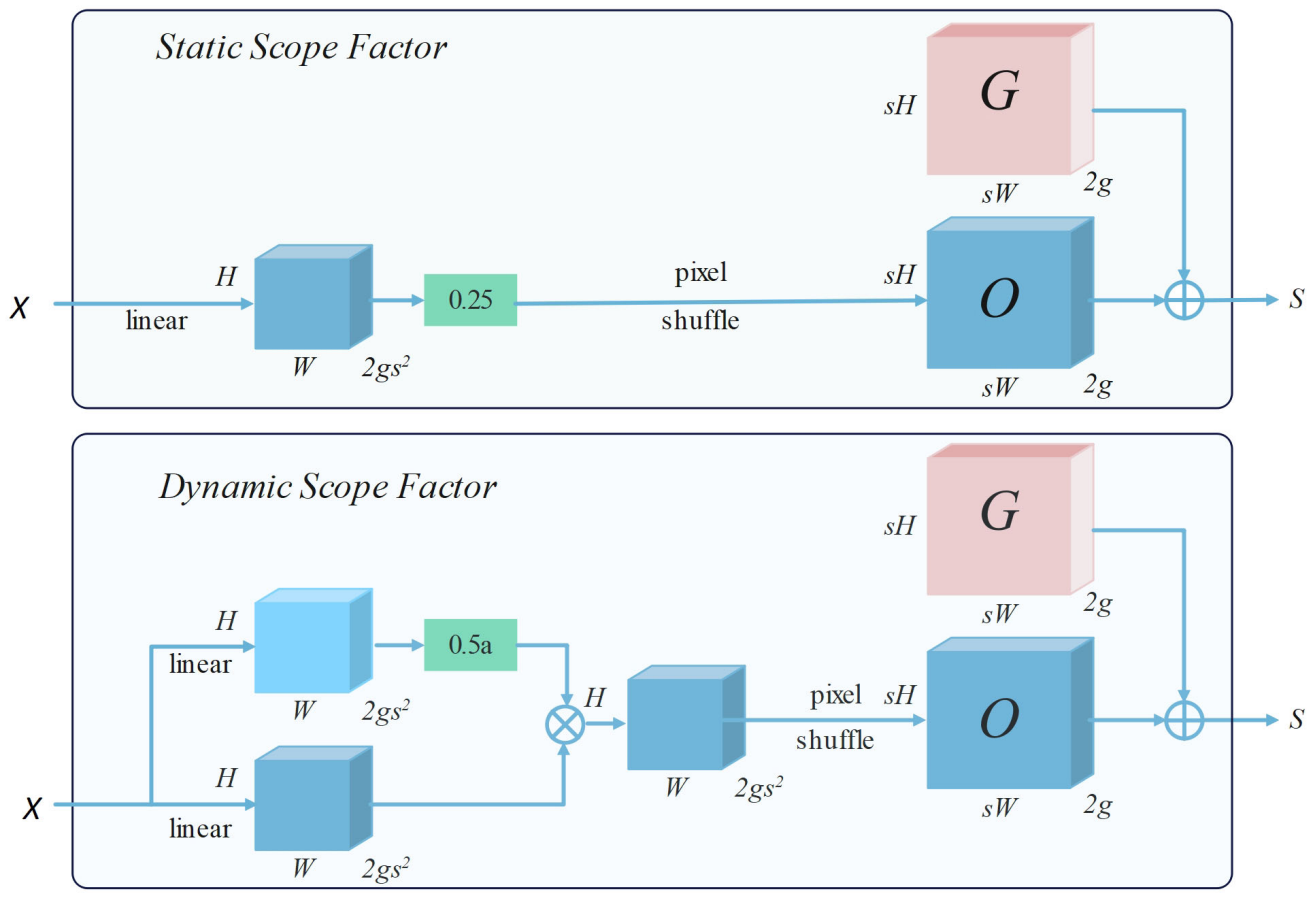
Process of point sampling data collection and generation.

The point sampling set S is generated in a manner of “linear + pixel recombination”, and the offset range can be determined by static and dynamic range factors, as depicted in **Figure 5**.

Taking the sampling method based on the static range factor as an example, given a feature map X of size C×H×W and an upsampling factor s. X first passes through a linear layer with input and output channels of C and 2gs^2^, and then uses the pixel recombination method to reshape it into an offset O of 2g×sH×sW, while the sampling set S is the sum of the offset O and the original sampling grid G. The specific calculation definitions are as follows (as shown in Equations 2, 3):


(2) O=Linear(X)

(3) S=G+O

This method dynamically adjusts each point of upsampling by learning offsets, thereby more accurately restoring the detailed features of rice diseases, enhancing the model’s perception of details, and enabling more precise localization and identification of rice diseases.

### 3.4 Optimization of the loss function

**Figure 6:**
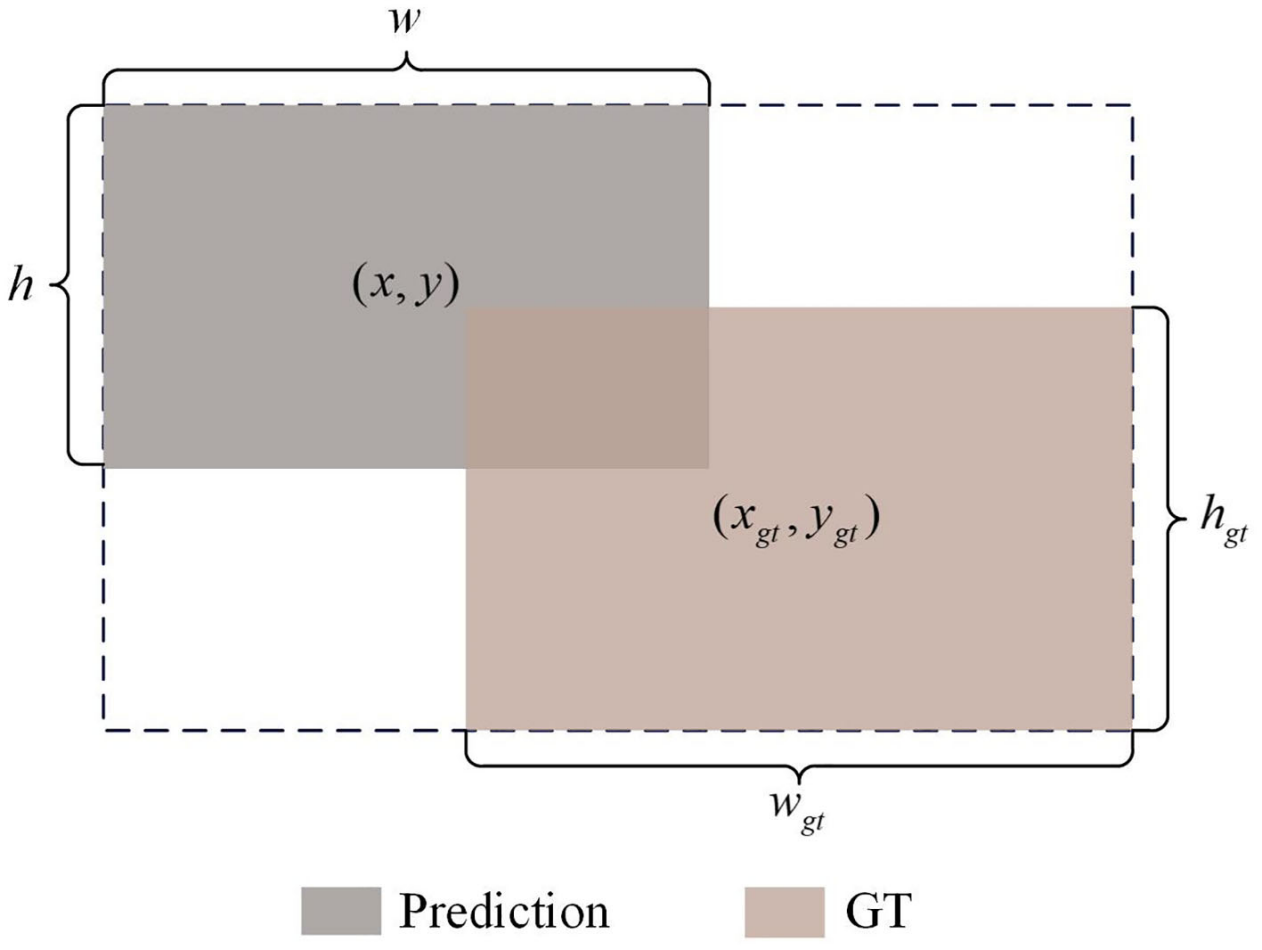
Illustration of Shape-IoU parameters.


YOLOv8 uses CIoU Loss to calculate the regression loss of bounding boxes. This loss function comprehensively considers the overlap area between the detection box and the target box, the loss being 0 when the bounding boxes do not intersect, the distance between the center points of the bounding boxes, and scale information such as the aspect ratio, thereby further improving the detection accuracy. However, CIoU Loss fails to fully consider the impact of the shape and size of the bounding box itself on the regression results. Based on this, we choose ShapeIoU (Zhang and Zhang, 2023) as the loss function for bounding box regression to focus on the shape and size characteristics of the bounding box. ShapeIoU can be derived from **Figure 6**, and the definition is as follows (as shown in Equations 4–7):

(4) $$L_{Shape-IoU}=1-L_{IoU}+distance^{shape}+0.5\times \Omega^{shape}$$

(5) $$L_{IoU}=\frac{\left|B\cap B_{gt}\right|}{\left|B\cup B_{gt}\right|}$$

(6) $$distance^{shape}=h_{a}\times \frac{{\left(x_{c}-x_{c}^{gt}\right)}^{2}}{c^{2}}+\omega_{b}\times \frac{{\left(y_{c}-y_{c}^{gt}\right)}^{2}}{c^{2}}$$

(7) $$\Omega^{shape}=\sum_{t=,h}{\left(1-e^{-w^{t}}\right)}^{\theta }$$

### 3.5 SSD-YOLO

**Figure 7:**
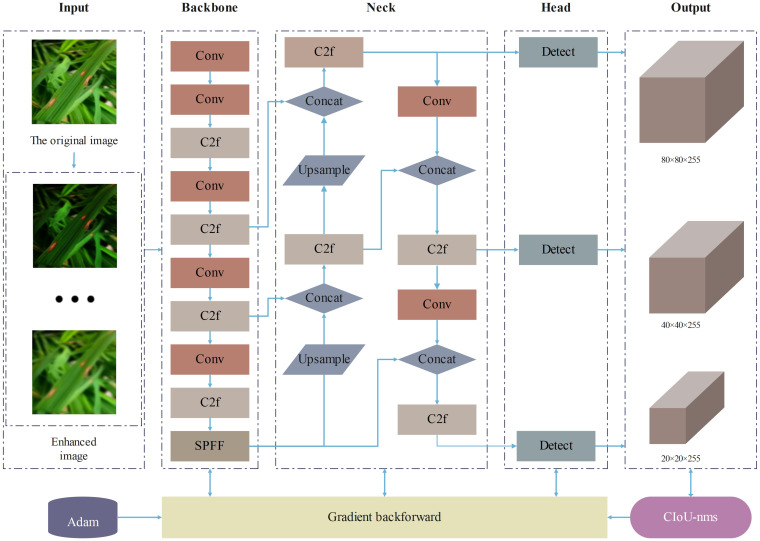
SSD-YOLO architecture diagram.

This study has implemented three improvements on the original YOLOv8 model, proposing an enhanced model named SSD-YOLO. The modified network structure is depicted in **Figure 7**. The specific improvements include:

1. The loss function CIoU in the original YOLOv8 is substituted with ShapeIoU. The updated loss function can generate higher-quality bounding boxes and significantly mitigate potential biases in the evaluation results.
2. SENet is incorporated into the enhanced feature fusion module C2F to strengthen the feature selection and the fusion capability of features from different channels, thereby achieving efficient computation and enhancing the model’s recognition ability of diseases in complex scenarios.
3. The DySample lightweight dynamic upsampling module is introduced, conducting upsampling from the perspective of point sampling. This approach effectively enhances the model’s anti-interference ability and, in contrast to the kernel-based dynamic upsampling module, demands fewer parameters, thereby conserving computing resources and being more applicable for real-time detection applications.

## 4 Analysis of experimental results

### 4.1 Rice leaf disease dataset

**Figure 8:**
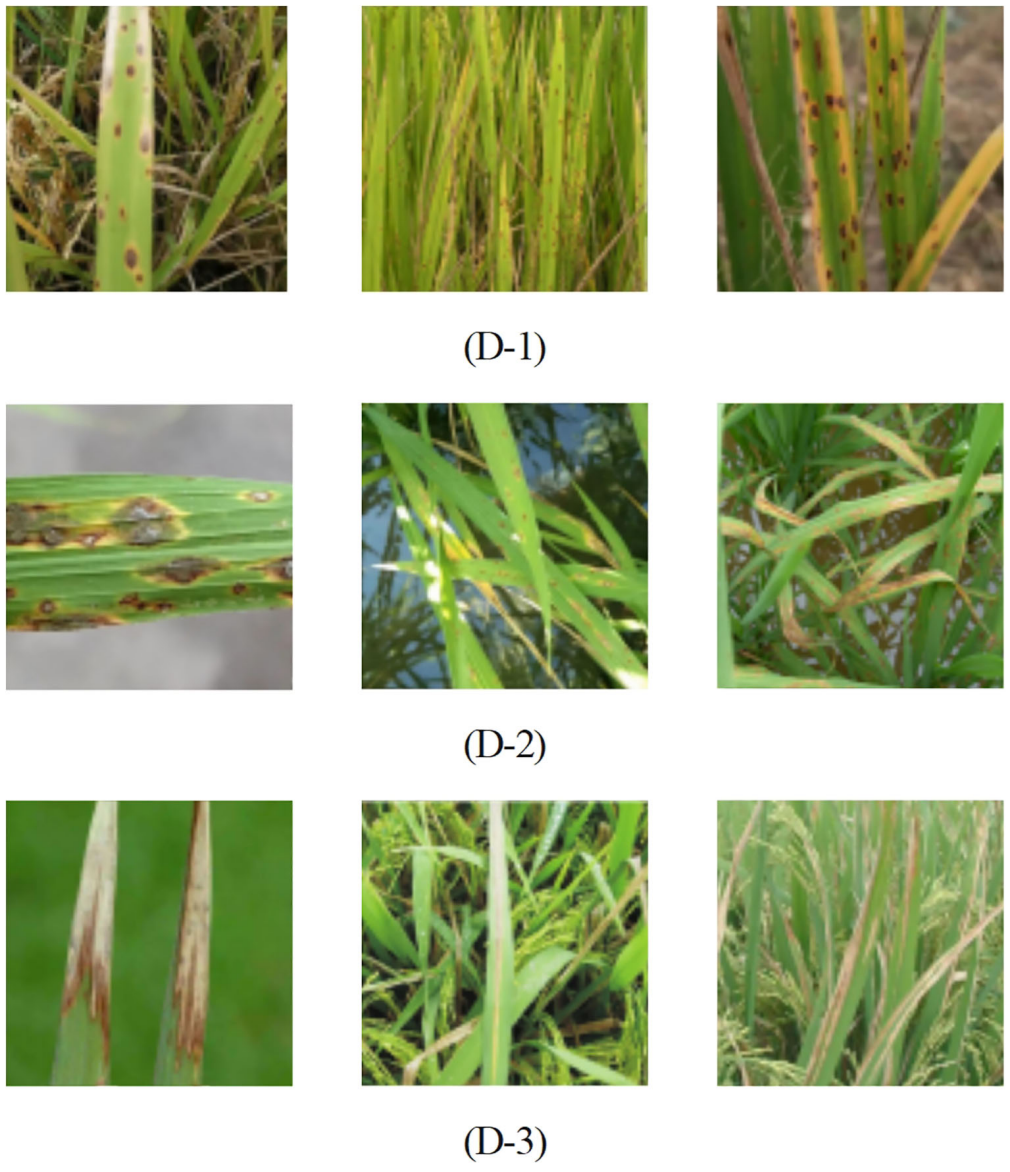
Common three types of rice diseases, (D-1) Rice Brown Spot, (D-2) Rice Blast, (D-3) Bacterial Leaf Blight.

To acquire a large-scale dataset of rice leaf disease images, this study developed an automated web crawler system utilizing the Selenium library. This system systematically collected and downloaded batches of images related to rice leaf diseases from the Internet. By employing specific keywords such as “rice leaf diseases” in major search engines including Baidu, Bing, and Google, we gathered a comprehensive dataset comprising 3,000 images. These images cover three primary types of rice leaf diseases: brown spot, blast, and bacterial blight. **Figure 8** illustrates the characteristic features of different rice leaf diseases.

The feature variations in rice leaf disease images are influenced by multiple factors, including shape, variety, and environmental conditions, leading to inconsistencies in resolution and contrast. To address these variations and ensure data consistency, we conducted preprocessing on the rice leaf disease dataset. First, we applied size normalization to standardize the spatial dimensions, enabling consistent comparisons across images. Second, we utilized image enhancement techniques such as histogram equalization and contrast stretching to adjust pixel value distributions, thereby enhancing the visibility of image details and edges. Finally, we simulated various weather conditions and time periods through digital processing to more accurately reflect real-world scenarios. These preprocessing steps enhanced the representativeness of the dataset and improved the model’s robustness and generalization capability in practical applications.

### 4.2 Evaluation metrics

This section delineates the comprehensive evaluation metrics utilized to rigorously assess the performance of the rice leaf diseases detection model. The key performance indicators encompass precision (P), recall (R), mean average precision (mAP), model size, and inference speed, which collectively provide a robust framework for model assessment.


(8) $$P=\frac{TP}{TP+FP}$$

(9) $$R=\frac{TP}{TP+FN}$$

(10) $$AP=\int_{0\;\;}^{1}\;P_{R}dR$$

(11) $$mAP=\frac{\int_{q=1}^{N}AP(q)}{N}$$

In Equations 8, 9, the calculation of precision (P) and recall (R) relies on three key indicators: true positives (TP), false positives (FP), and false negatives (FN). When the model successfully identifies rice leaf diseases, it is classified as TP; conversely, FP represents the number of incorrect detections of non-existent targets, while FN denotes the instances where actual targets are missed. Precision (P) assesses the model’s ability to accurately identify rice leaf diseases among all predicted targets, whereas recall (R) evaluates the proportion of successful identifications of actual targets by the model.

To evaluate detection performance for rice leaf diseases across different categories, a precision-recall (P-R) curve can be constructed. In Equations 10, 11, the average precision (AP) is defined as the area under this curve; thus, a higher AP value—closer to 1—indicates superior detection performance for that specific category. Mean Average Precision (MAP) refers to the weighted average of AP values across all categories and serves as a widely accepted metric for performance evaluation in object detection tasks. This metric offers both visualization and a comprehensive representation of overall model performance, with N in the equation denoting the total number of target categories.

Additionally, object detection speed is quantified in frames per second (FPS), where higher FPS values signify enhanced real-time processing capabilities. A thorough evaluation encompassing these indicators provides an exhaustive assessment of model performance, facilitating multidimensional comparisons and optimizations.

### 4.3 Experimental equipment

The experiment was conducted on a high-performance deep learning server equipped with two Nvidia RTX 3090 graphics cards, each with 24GB of VRAM. The operating system was Windows 11. The implementation of this method was based on Pytorch 1.9. The details of the experimental setup are shown in **Table 2**.

**Table 2:** High-performance server configuration table.

Item	Specification
CPU	Intel^®^ Core i9-10920X
GPU	Nvidia RTX 3090 24GB
Memory	128GB
Hard disk drive storage space	2TB
Operating system	Windows11(64-bit)
Programming environment	Python 3.8.8, Cuda 11.7, torch 1.9.1, torchaudio 0.9.1, torchvision 0.10.1

To optimize the network parameters, we utilized the Adam optimizer during the training of the SSD-YOLO model. To enhance convergence speed and ensure stable training, we set the initial learning rate (lr0) to 0.001 and gradually decayed it to a final learning rate (lrf) of 0.001. The loss function employed was ShapeIoU, with a momentum parameter of 0.937 and a weight decay of 0.0056. A warm-up strategy was implemented, where the learning rate started at a lower value for the first 5 epochs before gradually increasing to adapt to the data’s feature changes. Additionally, an early stopping mechanism was introduced to prevent overfitting and unnecessary training. Specifically, if the model’s performance did not improve significantly over 300 consecutive epochs, training would be halted. This approach ensures timely detection and intervention in case of suboptimal convergence, thereby maintaining the model’s optimal state.

### 4.4 Accuracy comparison of different attention mechanisms

The attention mechanism significantly enhances the model’s ability to capture key information in the input data by enabling the model to adaptively weight the importance of different positions or features in the sequence data. To systematically evaluate the impact of the SENetv2 attention mechanism on the performance of rice leaf disease recognition in the SSD-YOLO model, this study designed a comparative experiment to compare the performance of multiple mainstream attention mechanisms with the SSD-YOLO baseline model. Specifically, we selected four representative attention mechanisms for comparative analysis: (A) Context-aware attention mechanism, which dynamically adjusts feature weights based on global context relationships; (B) Multi-scale pyramid attention network, which captures cross-scale feature dependencies by constructing a hierarchical feature pyramid; (C) Residual attention bottleneck module, which innovatively combines residual connection with channel attention mechanism to achieve more refined feature optimization; (D) The improved SENetv2 attention mechanism proposed in this study. By comparing the experimental performance of these mechanisms, the effectiveness of different attention paradigms in disease identification tasks can be comprehensively evaluated.

**Table 3** shows the performance comparison results of different attention mechanisms on the yolov8 model. The experimental data show that after the introduction of attention mechanism, the model has achieved significant improvement in the two key indicators of map50 and map50-90. It is particularly noteworthy that the model using lightweight senetv2 attention mechanism has the most outstanding performance, with 94.3% and 77.1% excellent performance respectively. This result fully confirms the effectiveness of senetv2 attention mechanism in ssd-yolo architecture. Through the innovative lightweight design, the mechanism not only ensures the detection accuracy, but also significantly improves the computational efficiency and reduces the complexity of the model. This advantage of balancing performance and efficiency makes it particularly suitable for deployment in embedded devices or mobile terminals with limited computing resources.

**Table 3:** Experimental results of different attention mechanisms.

Model	Class	P (%)	R (%)	mAP50 (50%)	mAP50-90 (%)	Size (M)	FPS (frame/s)
YOLOv8n	All	91.0	84.4	89.7	64.8	5.99	46
	Brown spot	89.2	67.0	76.4	49.3		
	Rice blast	93.8	97.1	97.7	74.1		
	Bacterial Blight	90.1	89.1	95.2	71.1		
YOLOv8n+A	All	92.7	86.8	91.9	73.8	15.53	20
	Brown spot	88.6	71.7	81.3	54.1		
	Rice blast	94.9	97.6	98.1	83.2		
	Bacterial Blight	94.7	90.9	96.2	84.1		
YOLOv8n+B	All	95.8	87.9	93.1	76.5	6.02	38
	Brown spot	92.9	72.5	83.3	58.7		
	Rice blast	98.6	98.4	98.9	85.7		
	Bacterial Blight	95.9	92.7	97.1	85.1		
YOLOv8n+C	All	92.7	89.3	93.2	75.9	8.24	29
	Brown spot	85.8	75.3	83.6	58.0		
	Rice blast	96.1	98.8	98.5	84.3		
	Bacterial Blight	96.1	93.9	97.5	85.3		
YOLOv8n+D	All	94.7	90.3	94.3	77.1	6.05	40
	Brown spot	89.0	77.8	85.0	58.9		
	Rice blast	98.1	98.4	99.3	86.1		
	Bacterial Blight	96.9	94.7	98.6	86.4		

### 4.5 Heat map visualization

**Figure 9:**
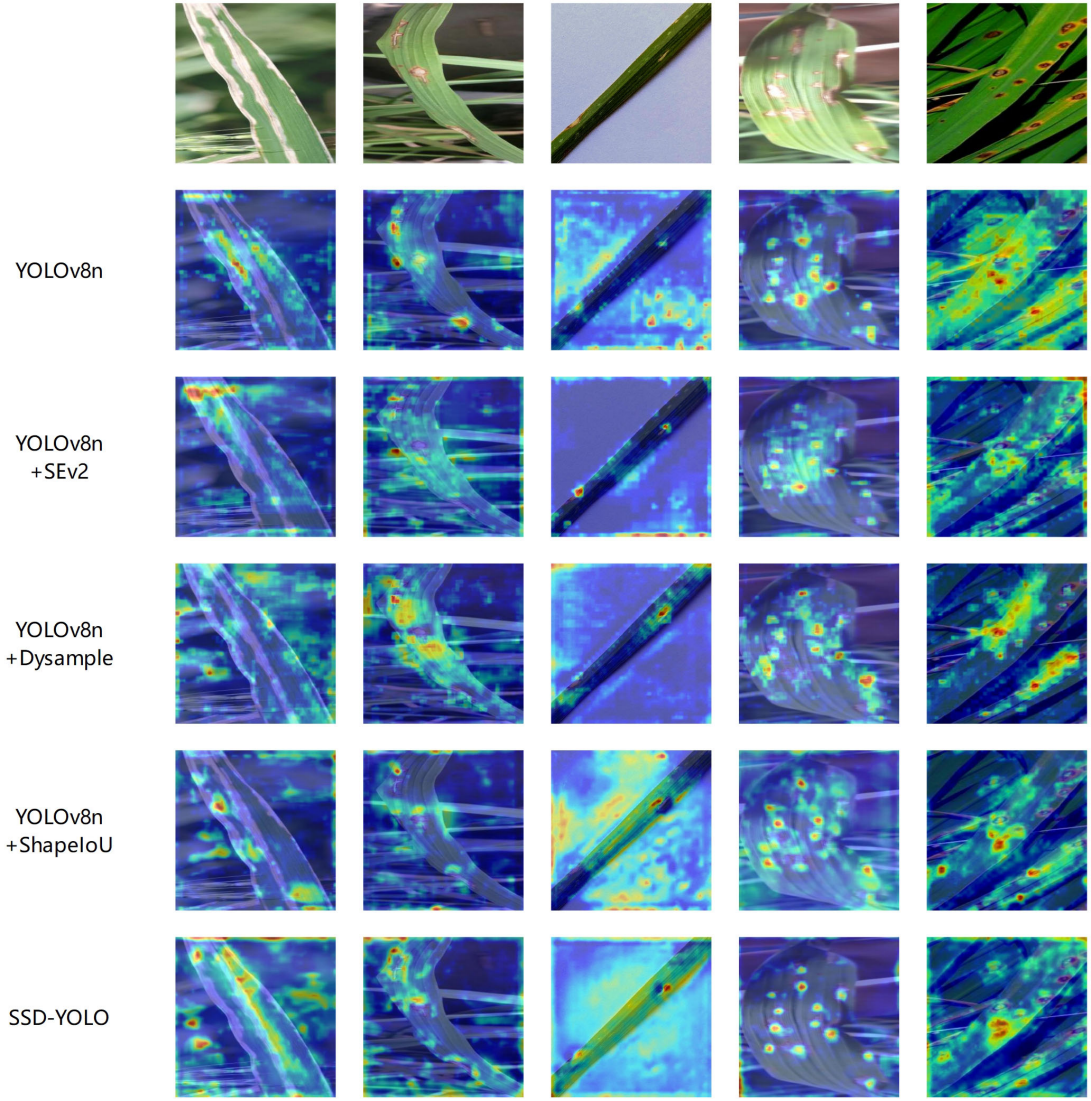
Heatmap experiment comparison.

This study employed Grad-CAM (Selvaraju et al., 2020) technology to construct a visualization heatmap for the detection of rice leaf diseases, providing an intuitive display of the target detection effect of rice leaf diseases and fully comparing the performance differences before and after the improvement of the YOLOv8n model. As shown in **Figure 9**, five groups of heatmap comparison results were generated. It can be clearly observed from the figure that the red high-confidence regions of the SSD-YOLO algorithm are more concentrated and highly consistent with the true center of the target, indicating that its positioning accuracy is significantly better than that of the original model and other comparison algorithms. This improvement mainly benefits from the following three technological innovations: Firstly, by introducing the SENet attention mechanism, the Bottleneck structure of YOLOv8 was optimized, significantly enhancing the model’s feature extraction capability. Secondly, the DySample lightweight dynamic upsampling module was adopted, specifically addressing the issue of high similarity between rice diseases and the background, further improving the model’s discrimination ability. Additionally, the ShapeIoU Loss was used to replace the traditional CIoU loss function, effectively enhancing the model’s detection performance in complex environments.

The visualization results fully confirm that the improved algorithm can achieve a dual advantage of detection accuracy and anti-interference ability in complex scenarios. The heatmap visualization experiment further verified that the model proposed in this study has higher accuracy and stronger feature extraction ability in feature extraction compared to the original model.

### 4.6 Analysis of detection results

The rice disease leaf detection model proposed in this study has been systematically verified under various environmental conditions and multi-object scenarios. The experimental results show that the model can not only accurately identify the leaves of rice with a single disease type and their background, but also maintain excellent detection performance in complex situations where multiple diseased leaves overlap and the disease feature areas have significant differences.

**Figure 10:**
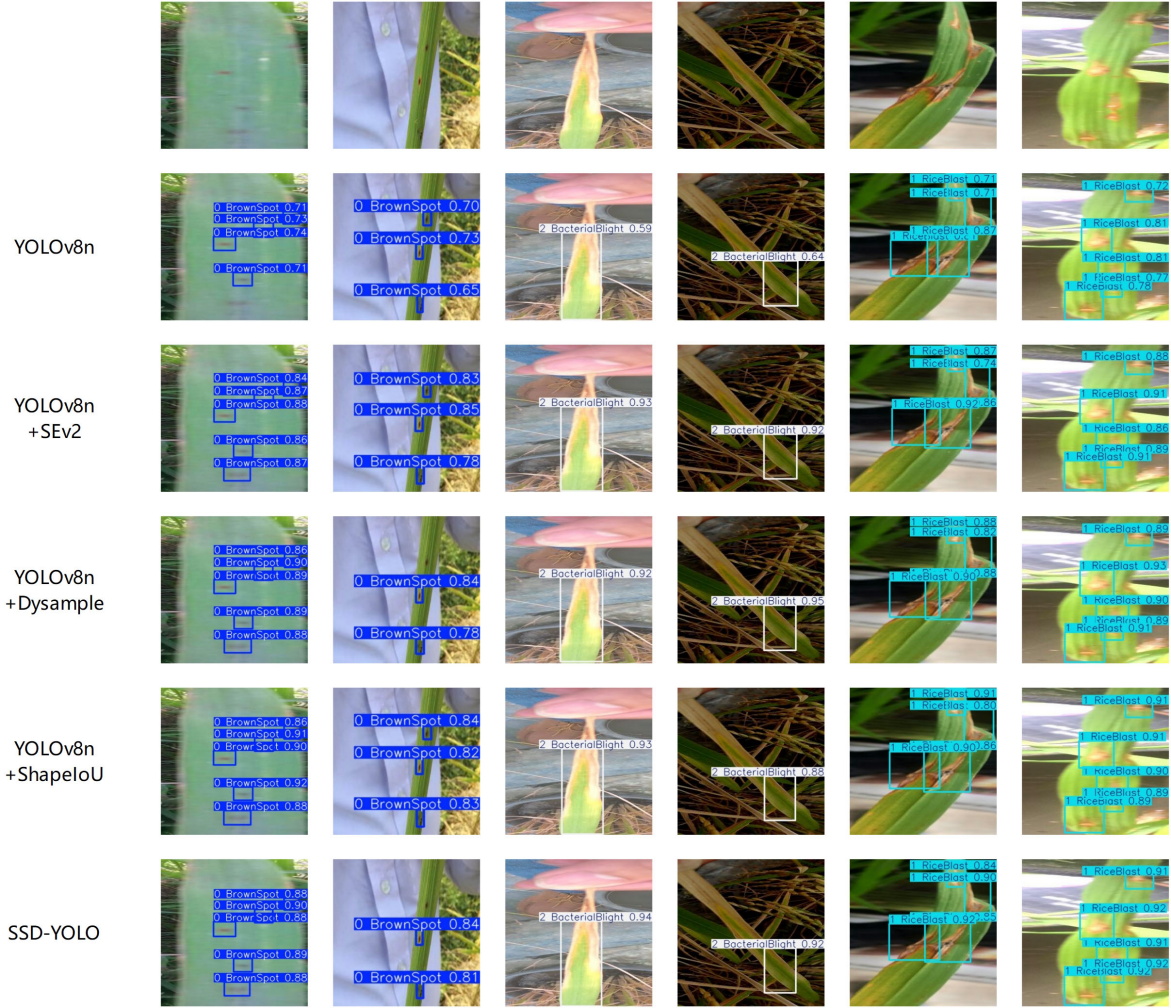
Detection result comparison.

To comprehensively evaluate the performance advantages of the proposed SSD-YOLO model in the identification of rice leaf lesions, this study designed and implemented a multi-model comparison experiment. Specifically, SSD-YOLO, the original YOLOv8n, an improved version using the ShapeIoU loss function, an optimized model incorporating the SE attention mechanism, and a variant model integrating the Dysample upsampling module were respectively applied to the test set images, and their detection results were thoroughly compared and analyzed. The relevant experimental results are shown in **Figure 10**, which fully verify the significant advantages of the SSD-YOLO model in the disease recognition task.

### 4.7 Comparison of the accuracy of different models

To objectively highlight the advantages of the SSD-YOLO model, this study conducted a systematic comparison with a two-stage object detection model (Faster R-CNN) and several one-stage object detection algorithms, including RT-DETR, YOLOv5 through YOLOv11,YOLOv8-DiDL (Guo et al., 2025), YOLOv8n-SMAFM (Jin et al., 2025), S-ZFFTNet (Muthusamy et al., 2025). The detailed results are presented in **Table 4**. This comparative analysis seeks to thoroughly evaluate the performance of different models in rice disease detection.

**Table 4:** Experimental results of different models.

Model	P (%)	mAP50 (50%)	Size (M)	FPS (frame/s)
Faster-RCNN	56.12	80.12	113.5	20
RT-DETR	75.67	70.56	63.1	24
YOLO v5	87.61	87.98	5.05	45
YOLO v6	88.62	85.9	8.31	41
YOLO v8	91.45	89.7	5.99	46
YOLO v9	90.22	92.45	14.5	26
YOLO v10	85.87	88.1	5.51	33
YOLO v11	91.32	89.28	5.25	43
YOLOv8-DiDL	91.4	90.8	4.8	55
YOLOv8n-SMAF	85.1	83.7	3.37	59
S-ZFFTNet	92.65	90.86	9.85	43
SSD-YOLO	95.19	95.32	6.08	50

According to the data presented in **Table 4**, the SSD-YOLO model proposed for rice disease detection demonstrates superior performance in identifying diseases from images. The mAP and accuracy of SSD-YOLO reached 95.32% and 95.19%, respectively, representing improvements of 5.51% and 3.74% over the baseline model YOLOv8. Compared with other state-of-the-art algorithms, SSD-YOLO achieved higher mAP values than RT-DETR, YOLOv5, YOLOv6, YOLOv9, YOLOv10, YOLOv11, YOLOv8-DiDL, YOLOv8n-SMAFM and S-ZFFTNet by 15.2%, 24.76%, 7.34%, 9.42%, 2.87%, 6.04%,4.52%,11.62% and 4.46%, respectively. In addition to evaluating average accuracy, frames per second (FPS) is a critical performance metric that reflects the number of target detection frames processed per second. SSD-YOLO exhibits a notable detection speed, making it suitable for most real-time applications. Moreover, SSD-YOLO not only boasts a smaller model size but also maintains an efficient detection speed, enhancing its practicality in resource-constrained. environments.

### 4.8 Ablation study

To validate the individual contribution of each new module in SSD-YOLO to the overall performance, we conducted a systematic ablation analysis on the rice disease dataset. The results of all ablation experiments are detailed in **Table 5**. Specifically, “A” denotes the addition of SENetv2 attention, “B” represents the adoption of the DySample sampling module, and “C” signifies the use of ShapeIoU as the loss function. The final model incorporates all these enhancements.

**Table 5:** Ablation study results.

Model	Class	P (%)	R (%)	mAP50 (50%)	mAP50-90 (%)	Size (M)	FPS (frame/s)
YOLOv8n	All	91.0	84.4	89.7	64.8	5.99	46
	Brown spot	89.2	67.0	76.4	49.3		
	Rice blast	93.8	97.1	97.7	74.1		
	Bacterial Blight	90.0	89.1	95.2	71.1		
YOLOv8n+A	All	94.7	90.3	94.3	77.1	6.05	40
	Brown spot	89.0	77.8	85.0	58.9		
	Rice blast	98.1	98.4	99.3	86.1		
	Bacterial Blight	96.9	94.7	98.6	86.4		
YOLOv8n+B	All	96.1	88.8	94.1	79.9	6.01	43
	Brown spot	91.9	74.9	85.0	62.0		
	Rice blast	98.9	98.8	99.0	87.6		
	Bacterial Blight	97.5	92.7	98.5	90.1		
YOLOv8n+C	All	96.1	89.6	94.2	80.1	6.31	45
	Brown spot	91.7	76.7	85.2	61.8		
	Rice blast	98.6	98.8	98.8	88.3		
	Bacterial Blight	98.1	93.3	98.6	90.1		
YOLOv8n+A+B	All	94.6	90.8	93.7	77.8	6.08	41
	Brown spot	88.7	79.2	85.5	60.1		
	Rice blast	96.9	98.8	98.5	87.1		
	Bacterial Blight	98.1	94.3	97.1	86.1		
YOLOv8n+A+C	All	93.2	91.1	93.7	78.2	6.08	39
	Brown spot	86.3	80.2	85.9	60.1		
	Rice blast	98.0	98.7	98.4	87.2		
	Bacterial Blight	95.1	94.6	96.8	87.2		
YOLOv8n+B+C	All	93.3	89.8	93.3	75.1	6.02	38
	Brown spot	88.8	77.0	83.7	59.7		
	Rice blast	96.9	98.6	98.9	82.3		
	Bacterial Blight	94.2	93.9	97.2	83.2		
SSD-YOLO	All	95.1	90.7	95.3	78.7	6.08	50
	Brown spot	89.9	78.8	87.5	61.7		
	Rice blast	97.3	98.8	99.4	87.0		
	Bacterial Blight	98.1	94.5	98.9	87.5		

Based on the analysis of the experimental results, it can be concluded that in the task of identifying rice leaf diseases, the combination of SENetv2, ShapeIoU bounding box regression loss function, and DySample module significantly enhances the overall performance of the model. Compared with YOLOv8, the SSD-YOLOv8 algorithm achieves improvements of 3.74% in accuracy, 7.0% in recall rate, and 5.51% in mAP. The integrated application of these strategies not only boosts the precision of rice disease detection but also enhances target localization, thereby providing more robust support for practical applications.

## 5 Conclusion

This study introduces SSD-YOLO, an enhanced model based on YOLOv8 for the detection of rice leaf diseases. The model integrates SENetV2 to optimize feature extraction, employs DySample for lightweight upsampling, and utilizes ShapeIoU for precise localization. Experimental results indicate significant improvements in performance, achieving detection accuracies of 87.52%, 99.48%, and 98.99% for brown spot, blast, and bacterial blight respectively, while maintaining a compact model size of just 6MB.

Despite these advancements, SSD-YOLO exhibits notable limitations: (1) Generalization to highly variable field conditions—such as variations in lighting and occlusions—remains a challenge; (2) The model’s dependence on high-quality training data may limit its applicability in resource-constrained regions where datasets are sparse.

Future work will focus on deploying SSD-YOLO in large-scale rice farms through partnerships with agricultural technology companies. By integrating the model with autonomous drones and soil sensors, we aim to build a closed-loop digital agriculture system that not only detects diseases but also recommends optimal treatment strategies based on historical data and environmental factors.

## Data Availability

The raw data supporting the conclusions of this article will be made available by the authors, without undue reservation.
